# The Ubiquitination of the Influenza A Virus PB1-F2 Protein Is Crucial for Its Biological Function

**DOI:** 10.1371/journal.pone.0118477

**Published:** 2015-04-13

**Authors:** Ivan Košík, Margaréta Práznovská, Martina Košíková, Zuzana Bobišová, Jaroslav Hollý, Eva Varečková, František Kostolanský, Gustáv Russ

**Affiliations:** Institute of Virology, Slovak Academy of Sciences, Dúbravská cesta 9, 845 05, Bratislava, Slovak Republic; Centers for Disease Control and Prevention, UNITED STATES

## Abstract

The aim of the present study was to identify what influences the short half-life of the influenza A virus PB1-F2 protein and whether a prolonged half-life affects the properties of this molecule. We hypothesized that the short half-life of PB1-F2 could conceal the phenotype of the protein. Because proteasome degradation might be involved in PB1-F2 degradation, we focused on ubiquitination, a common label for proteasome targeting. A cluster of lysine residues was demonstrated as an ubiquitination acceptor site in evolutionary and functionally distinct proteins. The PB1-F2 sequence alignment revealed a cluster of lysines on the carboxy terminal end of PB1-F2 in almost all of the GenBank sequences available to date. Using a proximity ligation assay, we identified ubiquitination as a novel posttranslational modification of PB1-F2. Changing the lysines at positions 73, 78, and 85 to arginines suppressed the ubiquitination of A/Puerto Rico/8/1934 (H1N1)-derived PB1-F2. The mutation of the C-terminal lysine residue cluster positively affected the overall expression levels of avian A/Honk Kong/156/1997 (H5N1)- and mammalian A/Puerto Rico/8/1934 (H1N1)-derived PB1-F2. Moreover, increased PB1-F2 copy numbers strengthened the functions of this virus in the infected cells. The results of a minigenome luciferase reporter assay revealed an enhancement of viral RNA-dependent RNA polymerase activity in the presence of stabilized PB1-F2, regardless of viral origin. IFNβ antagonism was enhanced in 293T cells transfected with a plasmid expressing stabilized K→R mutant variants of PB1-F2. Compared with PB1-F2 wt, the loss of ubiquitination enhanced the antibody response after DNA vaccination. In summary, we revealed that PB1-F2 is an ubiquitinated IAV protein, and this posttranslational modification plays a central role in the regulation of the biological functions of this protein.

## Introduction

In addition to humans, the influenza A virus (IAV) infects other species, including birds. IAV sporadically crosses the host species barrier, rapidly adapts to human hosts, thereby leading to global pandemics. More than half a million individuals succumb to IAV infections per year [1]. As IAV possesses a relatively small genome, differential mechanisms have evolved to increase the coding capacity of the segmented 13 kb (-)ssRNA genome, including alternative splicing (NEP, M2, M42), ribosomal frame shift (PB1-F2, PB1-N40, PA-X) or alternative ATG in-frame translation initiation (PA-N155, PA-N182). In the last 15 years, the IAV proteome has increased from 10 to 16 proteins [2]. The first of these “novel” proteins, PB1-F2, was identified in 2001 [3]. Interactions with other IAV proteins, i.e., the PB1-F2 to PB1 interaction mediated through the N-terminal domain of PB1-F2 [4, 5], are most likely responsible for the regulation of viral RNA-dependent RNA polymerase (vRdRp) activity. The enhancement of vRdRp activity is virus strain- and host cell-dependent [6], and the the amino acids at positions 51, 56 and 87 of the PB1-F2 ORF are particularly important for this regulation [7]. Although the PB1-F2 proteins derived from some isolates exhibit only minor effects, the enhancement of vRdRp activity has been observed for PB1-F2 from other strains and host cells [6, 8]. Mammalian isolate-derived PB1-F2 possesses a pro-apoptotic function likely associated with mitochondrial localization and interactions with anion nucleotide transporters and voltage anion-dependent channels [9–11]. Notably, the pro-apoptotic effects of PR8-derived PB1-F2 proteins are only observed on immune-competent cells [12]. PB1-F2 has been demonstrated as a potent inducer of pro-inflammatory cytokines, such as IL-1β, TNF-α, IL-6, and CXCL1/KC (a major neutrophil chemoattractant) [13–15]. Several studies have shown evidence of the PB1-F2 antagonism of IFN-β [13, 16–18]. The PB1-F2-mediated modulation of the cytokine response increases the cellularity of the lungs and immunopathology of IAV [19, 20]. The *in vitro* replication of viruses possessing the full-length PB1-F2 ORF did not differ from that of PB1-F2 ORF-deficient mutants *in vitro*. However, it has been suggested that the presence of PB1-F2 ORF contributes to virus the pathogenicity, prolonged clearance [19, 21], inflammation and chemoattraction of immune competent cells in an *in vivo* mouse model [13, 22, 23]. Nevertheless, the expression level of PB1-F2 has not been examined or correlated with the pathogenicity of IAV. The host immune response to PB1-F2 could affect the biological function of this IAV protein. Although antibodies specific to PB1-F2 are induced in mice and humans [24, 25], and immunization with PB1-F2 confers some protection [26], PB1-F2 is considered as a weak humoral response immunogen. As immunogenicity is also dose-dependent, the increased expression of PB1-F2 could affect the immunogenic properties of PB1-F2. The enhanced induction of antibodies specific to PB1-F2 could be beneficial because antibodies could block the immunopathological effects of PB1-F2. The strain- and cell-dependent effects of PB1-F2 remain elusive. However, it was recently proposed that the presence of the full-length PB1-F2 ORF might not be predictive of PB1-F2 expression in cells infected with the influenza A virus, as downstream mRNA sequences could affect PB1-F2 expression levels [27].

PB1-F2 is coded on bi-cistronic mRNA that preferentially translates the PB1 protein. Rapid degradation also contributes to the low expression of PB1-F2. Previous studies have focused on posttranslational stability because modifications of the mRNA regulatory (Kozak) sequences or IAV promoter sequences could also affect PB1 expression and the amino acid sequence [28]. The aim of the present study was to characterize the properties responsible for the instability of PB1-F2. We established that PB1-F2 is targeted through posttranslational modification via ubiquitination of the lysine residues at positions 73, 78, and 85, representing a major ubiquitination cluster. Additionally, changing K to R in these positions dramatically affected the overall expression and expression kinetics of PB1-F2. Moreover, increased PB1-F2 expression affected or enhanced distinct functions, demonstrating that ubiquitination and instability are crucial biological features of PB1-F2.

## Materials and Methods

### Virus, cell lines and mice

A/PR/8/34 (H1N1) (a kind gift from Dr. Yewdell, NIH, USA) was propagated in embryonated chicken eggs. Madin-Darby Canine Kidney (MDCK) cells, and the A549 (human lung epithelial) and 293T (human embryonic kidney) cell lines were grown in Dulbecco’s Modified Eagle Medium (DMEM, BioWhittaker) supplemented with 5% FBS (HyClone) and 40 μg/ml gentamicin at 37°C and 5% CO_2_. The six-week-old female BALB/c mice used in this study were purchased from Masaryk University, Brno, Czech Republic.

### Ethical statement

In all experiments presented in this study, the animals were treated according to the approval and standards of the European Union and State Veterinary and Food Administration of the Slovak Republic. The State Veterinary and Food Administration of the Slovak Republic specifically approved this study (permission number 1002/11-221/3). The fundamental ethical principles and animal welfare requirements were respected.

### DNA immunization of the mice and euthanasia

Before i.m. DNA injection and tail vein blood collection, the mice were anesthetized i.p. with ketamine (140 mg/kg)/xylazine (5.55 mg/kg). For DNA vaccination, groups of mice (n = 14) were i.m. immunized with four doses of 50 μg of pDNA in 100 μl PBS at two-week intervals. For blood collection, the tail was placed into a 37°C water bath for 30 second, and 30 to 50 μl of blood was directly collected from the lateral vein. For final blood collection, the mice were anesthetized i.p. with ketamine (140 mg/kg)/xylazine (5.55 mg/kg) and rapidly decapitated under deep anesthesia using scissors.

### Antibodies

The PB1-F2 specific monoclonal antibody AG55, which recognizes the N-terminal region of the native and denatured PB1-F2 protein, has been previously described [26]. The rabbit polyclonal anti-UBQ affinity-isolated antibody was purchased in a buffered aqueous solution from Sigma-Aldrich (SAB4503053). The monoclonal anti-beta-actin antibody clone AC-15 was purchased as purified immunoglobulin in a buffered aqueous solution from Sigma-Aldrich (A1978). FITC-conjugated polyclonal goat anti-mouse IgG and HRP-conjugated polyclonal goat anti-mouse secondary antibodies were purchased from DAKO and used according to the manufacturer’s instructions. The Alexa fluor 790-conjugated goat anti-mouse IgG secondary antibody was purchased from Life Technologies and used according to the manufacturer’s instructions. Immune mouse serum (IMS) was collected at 2 weeks after the 2^nd^, 3^th^, and 4^th^ doses of DNA immunization.

### Plasmid constructs

The pEGFP-N1 (Clontech) plasmid was used as a transfection efficiency control. The construct pTriEx4 PB1-F2 expressing PR8-derived PB1-F2 under the control of the CMV promoter has been previously described [4]. In the present study, this construct is referred to as pPB1-F2 PR8. The plasmid pPB1-F2 Stop3aa, harboring a stop mutation in the PB1-F2 ORF, and the plasmid pHA, expressing PR8 (H1N1)-derived HA, have been previously described [26]. The construct expressing an ubiquitination-deficient K73/78/85R variant of PB1-F2, labeled pPB1-F2 PR8 3KR, was constructed using site-directed mutagenesis in two rounds. The Phusion Site-Directed Mutagenesis Kit (Thermo Scientific) was used for PCR mutagenesis. The primers were designed using the GenBank database sequence AC:EF467819 corresponding to the PR8 second gene segment. Two-step PCR mutagenesis was performed according to the manufacturer’s instructions. Primers K73, 85R-F: 5′ATGGAGGTTGTTCAGCAGACACGAGTAGACAAGCT3′ and K73, 85R-R: 5′CGTTTCAATACACGAGTTCTCAAAAATACCAGGATGG3′ were used in the first round of mutagenesis to generate K to R substitutions at positions 73 and 85 of the PB1-F2 ORF. The PCR product was separated using agarose gel electrophoresis, followed by gel extraction and purification using a gel extraction kit (Qiagen). The purified fragment was recircularized using T4 DNA ligase (Thermo Scientific) and subsequently transformed into *E*. *coli* XL1 competent cells. After sequence confirmation of the desired mutations, the construct was labeled pPB1-F2 PR8 K73, 85R and used as a template for the next round of mutagenesis with the primers K78R-F: 5′GAGAACTCGTGTATTGAGACGATGGAGGTTGTTCAG3′ and K78R-R: 5′AAAAATACCAGGATGGGTTGCCTCAAGGAAAGC3′. The section highlighted in gray indicates the introduced mutation. The PCR product was separated using agarose gel electrophoresis, followed by extraction, purification, recircularization, and transformation as described above. After sequence confirmation of the desired mutations, the construct was labeled pPB1-F2 PR8 3KR. PB1-F2 derived from A/Honk Kong/156/1997 (H5N1), GenBank AC:AF036362, was purchased (Proscience Tech) and cloned into the pTriEx4 vector. PCR amplification was performed using the primers PB1-F2 HK97-F: 5′GACGACGACAAGATGGAACAGGAACAGGATACACC3′ and PB1-F2 HK97-R: 5′TCAGCTTATCCATTCTCGTTTGCTCAGCTTATCCATTCTCGTTTGCT3′ and Kodex DNA polymerase (Novagen). The underlined primer sequence represents the overhang required for cloning into pTriEx EK/LIC (Novagen). The PCR-amplified DNA was separated by agarose gel electrophoresis and gel purified using a gel extraction kit (Qiagen). The DNA was treated with T4 DNA polymerase to generate ssDNA overhangs, followed by ligation to the linearized vector, and transformation into bacterial cells as described above. After sequence confirmation, the plasmid was labeled pPB1-F2 HK97. The construct expressing a K73/78/81/85R variant of PB1-F2 was generated using site-directed mutagenesis. Phusion Site-Directed Mutagenesis Kit (Thermo Scientific) was used for PCR mutagenesis. The primers were designed using the GenBank database sequence AC:AF036362. PCR amplification was performed using the primers PB1-F2 HK97 K8185R F: 5′ACGATGGAGGTTGTCCAGCAGACGAGAATGGATAAG3′ and PB1-F2 HK97 K7378R R: 5′CTCAAGACACGAGTTCTCAAAGAGTCCTGGG3′. The sections highlighted in gray indicate the introduced mutations. The PCR products were separated using agarose gel electrophoresis, followed by gel extraction, purification, recircularization, and transformation as described above. After sequence confirmation of the desired mutations (K73/78/81/85R), the construct was labeled pPB1-F2 HK97 4KR. Protein expression was confirmed using immunofluorescence. The RNA transcriptional plasmid pPolSapRib and PB1, PB2, PA, and NP expression plasmids, constructed on a pCDNA3.1+ backbone, were kindly gifted from Dr. Ervin Fodor (Oxford Molecular Pathology Institute) and Sir William Dunn (School of Pathology). The firefly luciferase reporter plasmid expressing panhandle ssRNA encoding the luciferase gene under the 5' and 3' URT of the PR8 matrix gene segment was constructed as follows. The plasmid pGL3basic (Promega) AC:U47295 was subjected to PCR mutagenesis using the primers Luc E271E-F: 5′GATTTGAAGAAGAACTGTTTCTGAGGAGC3′ and Luc E271E-R: 5′TATACATTAAGACGACTCGAAATCCACATATC3′ and the Phusion High-Fidelity PCR Master Mix (Thermo Scientific) to remove the SapI restriction site in the luciferase ORF (from 807 to 815 nucleotide). The PCR-amplified DNA fragment was separated by agarose gel electrophoresis and purified using a gel extraction kit (Qiagen). The recircularized pDNA was transformed into the bacterial strain XL1, and sequencing confirmed the loss of the SapI restriction site with no alterations to the amino acid sequence of the luciferase gene. To generate the firefly luciferase reporter plasmid, the SapI restriction site and 5' /3' UTR of the IAV M segment overhang were introduced using luciferase-specific primers. The SapI^-^ plasmid pGL3 basic (Promega) was used as the source of the luciferase gene. The PR8MLuc-F: 5′TAATGCTCTTCT*GCCAGCGAAAGCAGGTAGATATTGAAAG***ATGGAAGACGCCAAAAACATAAAG3′** and PR8MLuc-R: 5′TAATGCTCTTCC*ATTAGTAGAAACAAGGTAGTTTT***TTACACGGCGATCTTTCCG'** primers were used in an amplification reaction with Kodex DNA polymerase (Novagen) according to the manufacturer’s instructions. The underlined sequences represent the SapI restriction site, the italics represent the 5' or 3' UTR (promoter) of the PR8 M segment and the bold font represents the luciferase-specific sequence annealing to the vector. The PCR product and the pPolSapRib plasmid were digested with SapI (New England Biolabs), separated through agarose gel electrophoresis, and gel purified using a gel extraction kit (Qiagen). The purified PCR product and linearized pPolSapRib were ligated usingT4 DNA ligase (Thermo Scientific), followed by transformation into *E*. *coli* XL1 competent cells. Sequencing confirmed the correctness of the cloned plasmid and the presence of the IAV promoter and firefly luciferase ORF sequences in the correct orientation. The reporter construct was labeled pPolPR8MLuc. Luciferase expression in the presence of reconstituted vRdRp after co-transfection was confirmed as described in more detail below. The reporter plasmid expressing firefly luciferase under the control of human IFN-β promoter was constructed as follows. A total of 5 μg of pGL3basic DNA (Promega) was digested with restriction endonucleases XhoI and HindIII (Fermentas) according to the manufacturer’s instructions and subsequently incubated with 10U of the calf intestinal phosphatase (Fermentas) at 37°C for 30 min. The DNA fragment was separated through agarose gel electrophoresis and purified using a gel extraction kit (Qiagen). The human promoter sequence on GenBank sequence AC: EF064725 was predicted using the online web based tool Genomatix. The PCR amplification of the human IFN-β promoter from Human Genomic DNA: Male (Promega) was performed using Kodex DNA polymerase (Novagen) and primers hIFNB XhoI F: 5′CGGGCTCGAGTTTAAATCATCCCTAGATTACTTATAATAC3′ and hIFNB HindIII R: 5′TGCCAAGCTTGTTGACAACACGAACAGTGTCGCCTAC3′. The underlined sequences represent the restriction sites. The amplified human IFN-β promoter DNA fragment was digested with restriction endonucleases XhoI and HindIII (Fermentas), separated through agarose gel electrophoresis and purified using a gel extraction kit (Qiagen). The purified PCR product and linearized dephosphorylated pGL3basic were ligated using T4 DNA ligase (Thermo Scientific), followed by transformation into the *E*. *coli* XL1 competent cells. Sequencing confirmed the correctness of the cloned plasmid, and functionality was assessed after transfection into 293T cells and incubation with 25 μg/ml of polyinosinicpolycytidilic acid potassium salt (pIC) in a luciferase reporter assay as described in detail below. The construct was labeled pGLhIFNBpr. The plasmid pRLTK expressing renilla luciferase was used as a transfection efficiency normalization control.

### Immunofluorescence

MDCK cells were grown on glass coverslips. At 60–70% confluence, the cells were transfected with 1 μg of pDNA expressing the respective PB1-F2 protein variants using Turbofect *in vitro* transfection reagent (Thermo Scientific). At different times post-transfection (p.t., 3 to 20 hr), the cells were fixed with 3% paraformaldehyde (Sigma) in PBS for 10 min and subsequently permeabilized with 0.1% Triton X-100 (Koch-Light) for 5 min. The samples were washed twice with PBS for 5 min and incubated with the freeze-thawed MDCK lysate-preabsorbed anti-PB1-F2 mAb AG55 diluted in PBS (10 μg/ml) for 90 min at room temperature. The samples were subsequently washed as previously described, and the slides were incubated with goat anti-mouse secondary antibody conjugated to FITC (DAKO) at a 1:100 dilution for 1 hr. After washing, the samples were mounted with mounting medium (Santa Cruz Biotechnologies). In the case of mitochondria visualization, the cells were incubated with pre-warmed DMEM supplemented with Mitotracker RedCMXRos (Life Technologies) at a final concentration of 100 nmol.l^-1^ for 30 min at 37°C and 5% CO_2_. After excessive washing with pre-warmed PBS, the samples were fixed and treated as described above. Fluorescence was visualized with a LSM Zeiss 510 Meta confocal microscope.

### Immunofluorescence-proximity ligation assay (IF-PLA)

The two primary anti-mouse and anti-rabbit antibodies recognizing the target proteins PB1-F2 and UBQ were used. Species-specific secondary antibodies, called PLA probes, attached to unique short DNA strands, bind to the primary antibodies. When the PLA probes are in close proximity (<40 nm), the DNA strands interact through the subsequent addition of two other circle-forming DNA oligonucleotides. After joining the two added oligonucleotides through enzymatic ligation, the DNA was amplified via rolling circle amplification using a polymerase. After amplification, the circularized DNA was replicated several-hundred, and the product was fluorescently labeled with complementary oligonucleotide probes. The resulting high concentration of fluorescence in each single-molecule amplification product was visible as a distinct bright dot using a fluorescence microscope. This method facilitated the sensitive and specific detection of proteins, protein modifications or interactions *in situ* [29]. A modified PLA protocol was established for the simultaneous detection of PB1-F2 and PB1-F2UBQ. Briefly, MDCK cells were grown on glass coverslips to 60–70% confluence. The cells were transfected with 1 μg of pPB1-F2 PR8, pPB1-F2 PR8 3KR or pPB1-F2 Stop3aa DNA. At 24 hr p. t., the cells were fixed in 3% paraformaldehyde (Sigma) for 10 min and subsequently permeabilized using 0.1% Triton X-100 (Koch Light) for 5 min. A mixture of 1:100 diluted polyclonal rabbit anti-UBQ antibody (Sigma-Aldrich) and AG55 (10 μg/ml) diluted in the supplied antibody diluents was added to the samples for 90 min, followed by washing twice with solution A. At the PLA plus/minus the probe incubation step, secondary goat anti-mouse FITC-conjugated antibody (DAKO) was added at a 1:500 dilution. This modification facilitated the detection of PB1-F2 to correlate the transfection efficiency with *in situ* PB1-F2 ubiquitination detection. Subsequently, ligation and polymerization were performed as described in the standard protocol. The samples were mounted with the DAPI (4ʹ,6-diamidino-2-phenylindole)-containing medium supplied with kit. To exclude decreased efficiency of ubiquitination detection, the samples were processed using a standard procedure and subsequently compared to the modified protocol-processed samples, with no obvious difference observed. IF-PLA was visualized with a LSM Zeiss 510 Meta confocal microscope.

### Immunoblot analyses

293T cells were grown on 6-well plates to 60–70% confluence. Turbofect *in vitro* transfection reagent (Thermo Scientific) was used to transfect 2 ng of the pEGFP-N1 mixed with 4 μg of plasmids pPB1-F2 PR8, pPB1-F2 PR8 3KR, pPB1-F2 HK97, pPB1-F2 HK97 4KR, or pPB1-F2 Stop3aa. The pEGFP-N1 (4 μg) was transfected as an additional negative control. At 24 hr p.t., GFP fluorescence was examined in each well to confirm equal transfection efficiency, and the cells were directly lysed using P- M-PER Mammalian-protein extraction reagent (Thermo Scientific). The protein concentration was measured using a BCA protein assay kit (Thermo Scientific). The samples were mixed with 5X sample buffer, heated at 100°C for 10 min and centrifuged at 15000 g for 10 min. An equal amount of the protein samples (40 μg) was loaded and separated on a 12% polyacrylamide gel and subsequently transferred to a nitrocellulose membrane. The membrane was saturated with 1% nonfat dried milk in PBS supplemented with 0.1% Tween 20 for 90 min, washed 3 times for 10 min with PBS supplemented with 0.1% Tween 20, cut into slices and incubated 90 min to overnight in anti-PB1-F2 mAb AG55 diluted (10 μg/ml) in 1% nonfat dried milk in PBS supplemented with 0.1% Tween 20 or in anti-Beta-actin mAb AC-15 (Sigma-Aldrich) diluted 1:5000 in 1% nonfat dried milk in PBS supplemented with 0.1% Tween 20. After the washing, the membrane was incubated for 60 min in secondary goat anti-mouse Alexa 790-conjugated antibody (Life Technologies) diluted 1:10.000 in 1% nonfat dried milk with 0.1% Tween 20 in PBS. After washing 3 times for 10 min in 0.1% Tween 20 plus PBS, the membrane was scanned using the Odyssey CLx Infrared Imaging System (Li-cor) and analyzed using Image studio Lite software. To determine the PB1-F2 expression levels in human IFN-β promoter induction and vRdRp activity reporter assays, 5 μl of the replicates of a particular sample were pooled and subjected to the immunoblot procedure as described above. Alternatively, to detection PB1-F2 expression using specific antibodies in the IMS after DNA vaccination with pPB1-F2 PR8 or pPB1-F2 PR83KR, 500 μg of pB1-F2-transfected MDCK cell lysate was separated on a 12% polyacrylamide gel and transferred to a nitrocellulose membrane. The membrane was saturated with nonfat dried milk, washed in PBS, and cut into slices. The anti-PB1-F2 mAb AG55 was used to confirm PB1-F2 expression (data not shown). The respective IMS was diluted 1:100 in 1% nonfat dried milk in PBS and incubated with the membrane slices for 90 min. After washing, the membrane was incubated for 60 min in secondary goat anti-mouse HRP-conjugated antibody diluted 1:10.000 in 1% nonfat dried milk with 0.01% NP-40 in PBS. After washing 3 times for 10 min in PBS containing 0.01% NP-40, the membrane was developed using luminol solution (Sigma-Aldrich) and exposed to X-ray film.

### vRdRp reporter assay

The 293T cells were grown on 96-well plates. At 60–70% confluence, the cells were co-transfected with a mixture of the plasmids (50 ng per plasmid) pPB1, pPB2, pPA, pNP, pPolPR8MLuc and the respective PB1-F2 expressing plasmids (pPB1-F2 PR8/pPB1-F2 PR8 3KR/pPB1-F2 HK97/pPB1-F2 HK97 4KR/pPB1-F2 Stop3aa). Turbofect *in vitro* transfection reagent (Thermo Scientific) was according to the manufacturer’s instruction. At 48 hr p.t., the cells were gently washed with PBS and directly lysed in passive lysis buffer (Promega). Twenty microliters of the supernatant was transferred to an opaque, flat-bottom 96-well plate (Costar). A Synergy HT Multi-Mode Microplate Reader with automatic dispensers was used to add 50 μl LarII reagent (Promega). After a 2-second delay, the firefly luciferase luminescence in separate wells was measured. Subsequently, 50 μl of the Stop and Glo reagent (Promega) was added, and after a 2-second delay, the renilla luciferase luminescence in separate wells was measured. Three independent experiments were performed in triplicate for each sample. The luminescent signal for firefly luciferase was normalized to the renilla luciferase luminescent signal. The results were further normalized to the activity of vRdRp in the presence of the wt PB1-F2. The error bars indicate the SD for all independent measurements.

### IFN-β promoter induction luciferase reporter assay

The 293T cells were grown on 96-well plates. At 60–70% confluence, the cells were co-transfected with a mixture of the plasmids pGLhIFNBpr (100 ng), pRLTK (0.5 ng) and the respective PB1-F2 expressing plasmids (100 ng pPB1-F2 PR8/pPB1-F2 PR8 3KR/pPB1-F2 HK97/pPB1-F2 HK97 4KR/pPB1-F2 Stop3aa). At 24 hr p.t., the transfection medium was substituted with DMEM containing 0.5% FBS supplemented with potent IFN-β inducer pIC (25 μg/ml). At 15, 24 and 40 hr p.t., the supernatant was removed, and the cells were gently washed and directly lysed in passive lysis buffer (Promega). Twenty microliters of the supernatant was transferred to an opaque, flat-bottom 96-well plate (Costar). A Synergy HT Multi-Mode Microplate Reader with automatic dispensers was used to add 50 μl LarII reagent (Promega). After a 2-second delay, the firefly luciferase luminescence in separate wells was measured. Subsequently, 50 μl of the Stop and Glo reagent (Promega) was added, and after a 2-second delay, the renilla luciferase luminescence in separate wells was measured. Three independent experiments were performed in triplicate for each sample. The luminescent signal for firefly luciferase was normalized to the renilla luciferase luminescent signal. The results were further normalized to the induction of human IFN-β promoter in pPB1-F2 Stop3aa (NC)-transfected samples. The error bars indicate the SD for all of the independent measurements.

### Statistical and colocalization analysis

The statistical analysis was performed using GraphPad Prism software V5.00 (GraphPad Software Inc., San Diego, CA). One-way analysis of variance (ANOVA) with Tukey’s multiple comparisons was applied on IFN β promoter induction data after normalization to the renilla and firefly luciferase signals (measured in absence of PB1-F2). The same statistical analysis was applied to determine significant differences in the vRdRp activity data after normalization to renilla and firefly luciferase signals (measured in presence of the PB1-F2 PR8). The differences were considered statistically significant at *p*<0.05. Bitplane Imaris software was used to generate z stack colocalization 2D histograms and to determine Pearson’s coefficient in the colocalized volume calculation.

## Results and Discussion

### PB1-F2 is post-translationally modified through ubiquitin

**Fig 1:**
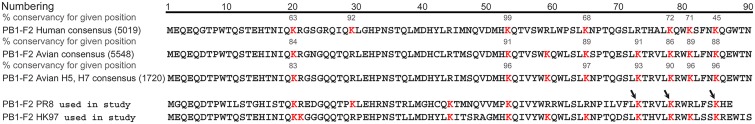
Comparison of the consensus sequences of PB1-F2 of Human, Avian, H5, H7 origin and sequences of PR8-, HK97- and 1918-derived PB1-F2. The consensus sequences of PB1-F2 from different species and the sequences of PB1-F2 from the model laboratory strains PR8 and HK97 were aligned, and all K residues are indicated in red to visualize differences in the potential ubiquitination sites. The numbers in brackets represent the amount of sequences used for consensus sequence generation. The numbers above the K residue represent the percentage of conservancy of the K residue for a given position in a set of analyzed sequences. The cluster of K residues is present in the C-terminal part of the molecule for human- and avian-derived PB1-F2.

**Fig 2:**
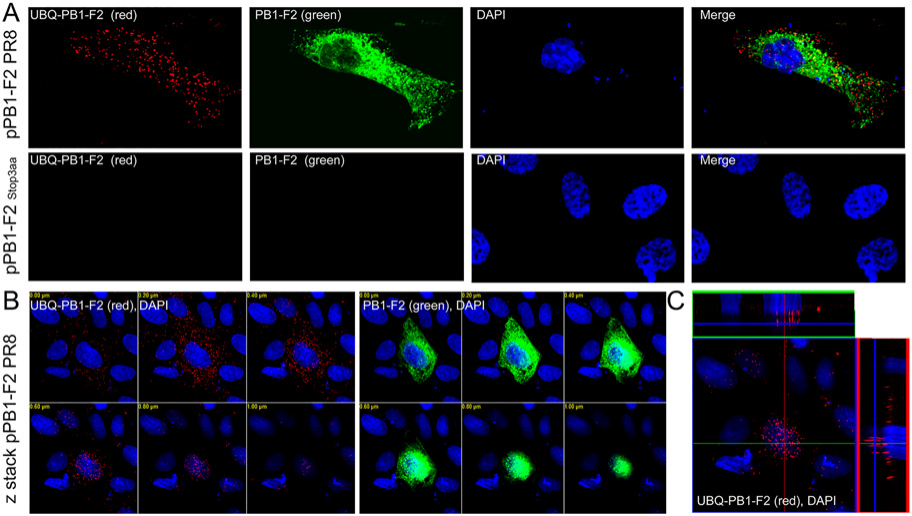
Detection of PB1-F2 ubiquitination using IF-PLA confocal microscopy. MDCK cells were transfected with 1 μg of pPB1-F2 PR8 or pPB1-F2 Stop3aa as a negative control. At 24 hr p.t., (A) the subpopulation of ubiquitinated PB1-F2 (labeled UBQ-PB1-F2, red spots, upper left panel) was detected using a mixture of primary antibodies specific to UBQ and PB1-F2 and the PLA probes. In the same cell, PB1-F2 (green) was stained with a secondary antibody conjugated with FITC. Neither PB1-F2 nor UBQ-PB1-F2 was detected in the MDCK cells transfected with pPB1-F2 Stop3aa (lower row). (B) Z stacking was used to visualize UBQ-PB1-F2 (below left panel) and PB1-F2 (below middle panel) in multiple focal planes to measure the overall extent of the expression, ubiquitination and localization of UBQ-PB1-F2 or PB1-F2. (C) Orthogonal section reconstruction was applied to observe the subcellular localization of UBQ-PB1-F2 (lower right panel). The nuclei were stained with DAPI.

Pioneering work on the discovery of PB1-F2 suggested the involvement of the proteasome machinery in the rapid degradation of PB1-F2, with a measured half-life of only 20 min. [3]. In general, proteins targeted for proteasome degradation are post-translationally labeled through the addition of ubiquitin (UBQ) [30]. With the rare exception of a serine or threonine, lysine residues have been identified as major ubiquitination sites [31, 32]. In the case of influenza virus, several proteins that interact with the ubiquitin-proteasome system (UPS) have been identified. Namely, PB2, PA and NP were shown to interact with cellular deubiquitinating enzyme USP11, and NP K148 was identified as a UBQ acceptor site [33]. Because PB1-F2 is synthesized on free ribosomes, it is logical to suspect the involvement of UPS in the short half-life of this protein. To measure the conservation of lysine residues in the PB1-F2 ORF, multiple sequence alignment was performed for human and avian isolates. A cluster of K residues was identified on the carboxy-terminal end of the molecule (Fig. 1). To establish the ubiquitination status of PR8-derived PB1-F2, we employed a modified immunofluorescence proximity ligation assay (IF-PLA). When primary antibodies bind to targets (PB1-F2, UBQ) in close proximity, the ligation and rolling cycle amplification of marker DNA conjugated to secondary antibodies occurs, and the hybridization of a fluorescently labeled probe provides a strong signal. As described in detail in the Material and Methods section, the PLA method facilitates the sensitive and specific detection of protein modifications *in situ*. MDCK cells were transiently transfected with pPB1-F2 PR8 or pPB1-F2 Stop3aa. At 24 hours post-transfection (hr p.t.), the ubiquitinated form of the PB1-F2 (UBQ-PB1-F2) was detected through PLA. The same sample was probed using conventional immunofluorescence for PB1-F2 expression (IF-PLA). The IF-PLA clearly demonstrated that PB1-F2-positive cells contained a subpopulation of ubiquitinated PB1-F2 protein (Fig. 2A). The red spots shown in Fig. 2 represent the ubiquitinated PB1-F2 molecules, and the green color represents the conventional detection of PB1-F2 using immunofluorescence. To observe the overall extent of ubiquitination and subcellular localization of UBQ-PB1-F2, confocal microscopy was employed. Different focal planes (z stacking) and orthogonal sectioning revealed that UBQ-PB1-F2 is extensively spread throughout the cytosol (Fig. 2B). Orthogonal sectioning clearly showed that UBQ-PB1-F2 is not present in the nucleus of PB1-F2-positive cells (Fig. 2C).

### Replacement of the C-terminal K residue cluster leads to the loss of PR8-derived PB1-F2 protein ubiquitination and enhances avian HK97-derived PB1-F2 nuclear localization, with no effect on mitochondrial localization

**Fig 3:**
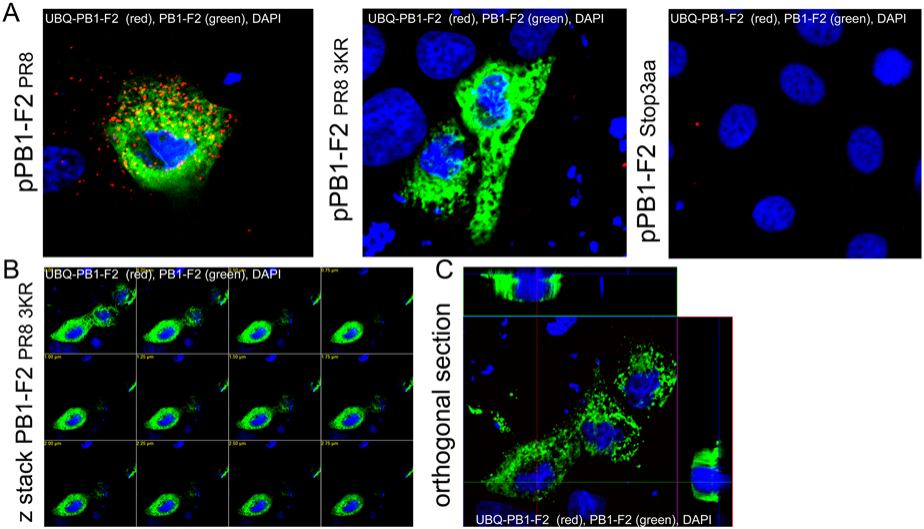
Identification of the cluster of C-terminal K residues as an ubiquitination site of PB1-F2. MDCK cells were transfected transiently with 1 μg pPB1-F2 PR8, pPB1-F2 PR8 3KR or pPB1-F2 Stop3aa. At 24 hr p.t., the cells were fixed and stained for the presence of the subpopulation of ubiquitinated PB1-F2 (labeled UBQ-PB1-F2, red spots) and PB1-F2 (green). (A) and analyzed with confocal microscopy. (B) Z stack acquisition was applied to confirm the absence of UBQ-PB1-F2 (red spots), regardless of the focal plane displayed. Orthogonal section reconstruction confirmed that the distribution of PB1-F2 PR8 3KR was equal to the wt PB1-F2 protein. (C) No UBQ-PB1-F2 (red spots) was observed in the cells transfected with pPB1-F2 PR8 3KR in multiple focal planes orthogonal section reconstruction. The nuclei were stained with DAPI.

**Fig 4:**
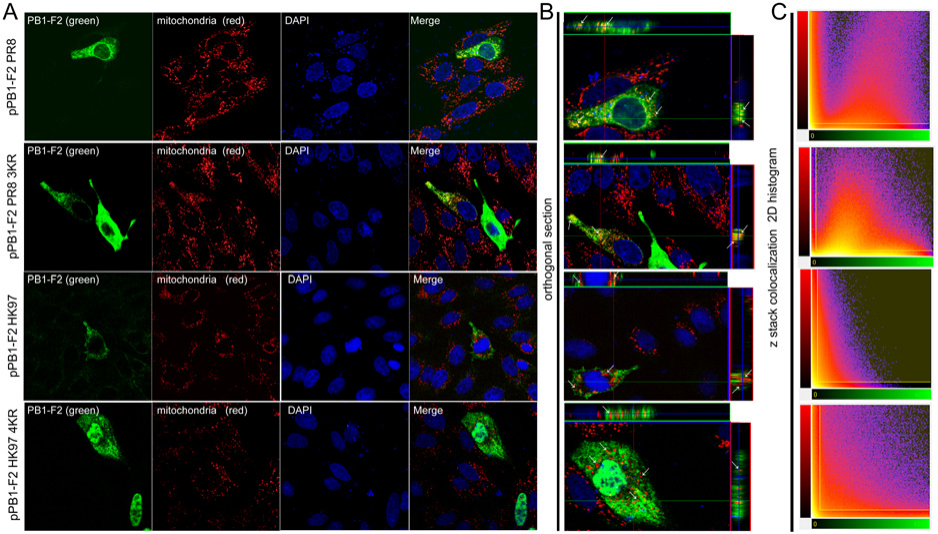
Subcellular localization of PR8- and HK97-derived PB1-F2 wt and the C-terminal K residue cluster mutants. MDCK cells were transfected transiently with the respective PB1-F2 expressing plasmid (1 μg pPB1-F2 PR8/pPB1-F2 PR8 3KR/pPB1-F2 HK97/ pPB1-F2 HK97 4KR). At 24 hr p.t., the cells were stained with Mitotracker RedCMXRos according to the manufacturer’s instructions (Life Technologies), fixed and stained for the presence of PB1-F2 variants (A). mAb AG55, specific to N-terminal region of the PB1-F2, and the FITC-conjugated secondary antibody were used to stain PB1-F2 (green, left column). Mitotracker RedCMXRos was used to stain mitochondria (red, second left column). Merge of PB1-F2 and mitochondrial signals (yellow, right column) showing clearly visible colocalization in PB1-F2 PR8 and PB1-F2 PR8 3KR-positive cells. The nuclei were stained with DAPI. (B) Orthogonal section reconstruction suggests the colocalization of the PB1-F2 with mitochondria signal throughout the entire cytoplasm for PR8-derived PB1-F2 and PB1-F2 PR8 3KR, but not for PB1-F2 HK97 and PB1-F2 HK97 4KR. The images were acquired at the same exposure, pinhole and optical slice conditions. (C) Image analysis software (Bitplane Imaris) was used to generate 2D histograms of the PB1-F2 (green) and mitochondria (red) signal intensities for volume pixels (voxels) and calculating Pearson’s coefficient in colocalized volume.

Next, we examined whether the cluster of PR8-derived PB1-F2 K73/78/85 is involved in PB1-F2 ubiquitination. To this end, we changed the C-terminal lysine residue cluster to arginines using site-directed mutagenesis. The changes were confirmed through sequencing, and the expression plasmid was referred to as pPB1-F2 PR8 3KR. MDCK cells were transfected with pPB1-F2 PR8, pPB1-F2 PR8 3KR or pPB1-F2 Stop3aa. As expected, IF-PLA confirmed that changing K 73, 78, and 85 to R lead to the loss of pPB1-F2 ubiquitination in PR8 3KR-transfected cells, while no change in UBQ status was detected in pPB1-F2 PR8-transfected cells (Fig. 3). Importantly, z-stacking and orthogonal section reconstruction confirmed the loss of PB1-F2 PR8 3KR ubiquitination in PB1-F2-positive cells (Fig. 3B, C). Ubiquitination has been associated with the alternation of the subcellular localization of various proteins [34–37]. Nevertheless, we did not observe any obvious change in PB1-F2 distribution associated with the loss of ubiquitination. To extend this observation, PB1-F2 derived from avian influenza virus A/Hong Kong/156/1997 (H5N1) (HK97) and the mutant K73/78/81/85R were employed. Previous studies have shown that PR8-derived PB1-F2 is preferentially localized to mitochondria, while HK97-derived PB1-F2 is spread throughout the entire cell [8]. To assess the effect of the K residue cluster change on the subcellular distribution of PB1-F2, MDCK cells were transiently transfected with pPB1-F2 PR8, pPB1-F2 PR8 3KR, pPB1-F2 HK97 or pPB1-F2 HK97 4KR. At 24 hr p.t., the cells were stained with Mitotracker RedCMXRos and subjected to immunofluorescence analysis to determine the subcellular localization of PB1-F2 protein. (Fig. 4) Mutation of the PB1-F2 protein C-terminal K residue cluster to R did not effect the mitochondrial localization of PR8- nor HK97-derived PB1-F2. Consistent with the results of previous studies [3, 6], PR8-derived PB1-F2 and the ubiquitination-deficient K73/78/85R mutant were predominantly detected in cytoplasm (Fig. 4A) and colocalized with mitochondria (Fig. 4B). The colocalization analysis (Bitplane Imaris software) of confocal microscopy z-stack data confirmed equal colocalization (Fig. 4C) with Pearson’s coefficient in colocalized volume (PCCV) PCCV = 0.035 for PB1-F2 (PR8) and PCCV = 0.0295 for PB1-F2 3KR (PR8). Comparison of the subcellular localization of the HK97-derived PB1-F2 and the K73/78/81/85R mutant variant showed the enhanced nuclear localization of PB1-F2 HK97 4KR. The ratio of nuclear PB1-F2 HK97 and PB1-F2 HK97 4KR was calculated by eye using an epifluorescence microscope after counting 400 to 500 positive cells on each coverslip examined. In contrast with previously published data suggesting the whole cell distribution of PB1-F2 HK97 [8], we observed preferential cytoplasmic localization, with only 38,5% nuclear localization. For PB1-F2 HK97 4KR, 73,4% positive cells exhibited preferential nuclear distribution. Notably, the immunofluorescence analysis revealed a significantly higher frequency of PB1-F2HK97 4KR-positive cells and a higher signal acquired under the same measurement conditions (Fig. 4A), suggesting the increased expression of PB1-F2 HK97 4KR and PB1-F2 PR8 3KR. Because PB1-F2 HK97 is also localized in the nucleus, and the expression of the PB1-F2 HK97 4KR is significantly higher, it is reasonable to suggest that increased nuclear localization frequency results from a higher protein concentration rather than the mutations. Consistent with previously published data [38], orthogonal sectioning (Fig. 4B) suggested that neither PB1-F2 HK97 nor PB1-F2 HK97 4KR colocalized with mitochondria, regardless of the high levels of PB1-F2 HK97 4KR expression. The colocalization analysis (Bitplane Imaris software) of the z-stack data obtained from confocal microscopy revealed the negligible colocalization of mitochondria and PB1-F2 HK97or PB1-F2 HK97 4KR (Fig. 4C). Despite high mitochondrial and PB1-F2 signals in cells transfected with pPB1-F2 HK97 4KR, the results of the z stack 2D histogram (Fig. 4C) support the negligible colocalization of PB1-F2 HK97, with PCCV = -0.0911, and PB1-F2 HK97 4KR, with PCCV = -0.0692, thereby confirming our hypotheses. The sequence alignment of more than ten thousand human and avian PB1-F2 sequences (Fig. 1) revealed a high degree of K cluster conservation. There are no viruses encoding a full-length PB1-F2 ORF lacking the K residue cluster on the C-terminal part of the molecule. The evolution of IAV has occasionally been associated with PB1-F2 ORF truncation from the C-terminus. Interestingly truncation preserves the lysine residue at position 53 on the C-terminus of the PB1-F2 (57 aa long) [8, 39]. Although viruses harboring full-length or truncated PB1-F2 variants do not differ significantly [40], there is no information regarding the expression of the full-length versus truncated PB1-F2. However, the presence of a C-terminal K in the truncated ORF could contribute to the ubiquitination of PB1-F2 when an altered conformation occurs. During the evolution of the second gene segment of IAV, at least two K residues were preserved in the PB1-F2 ORF. The amino acid sequence alignment of the human consensus and PR8-derived PB1-F2 ORFs (Fig. 1) showed variation in a single K residue position among these proteins. Seventy-one percent of human isolates contain a K residue at position 81, but when this residue is absent, i.e., PR8-derived PB1-F2, the missing K is compensated by an additional K residue at position 73 (common for 91% of avian-origin PB1-F2 proteins). The cluster of K residues in PB1-F2 is freely shifted from amino acid 77 to the end of the PB1-F2 ORF. Consistent with this observation, the accumulation of proximal lysines is preferentially targeted by UBQ [41]. Notably, no single K ubiquitination, but rather the entire C-terminal cluster of ubiquitinated lysine residues, is important for the biological behavior of the Neuronal Glycine Transporter [42, 43]. A K residue cluster has also been identified in the major UBQ acceptor site of Arn1p, a ferrichrome transporter in *Saccharomyces cerevisiae* [44]. We suggest that no single K residue at particular positions but rather the entire K cluster is important for the ubiquitination of PB1-F2.

### Replacement of the C-terminal K residue cluster of the PR8- and avian HK97-derived PB1-F2 protein with R positively affect expression

**Fig 5:**
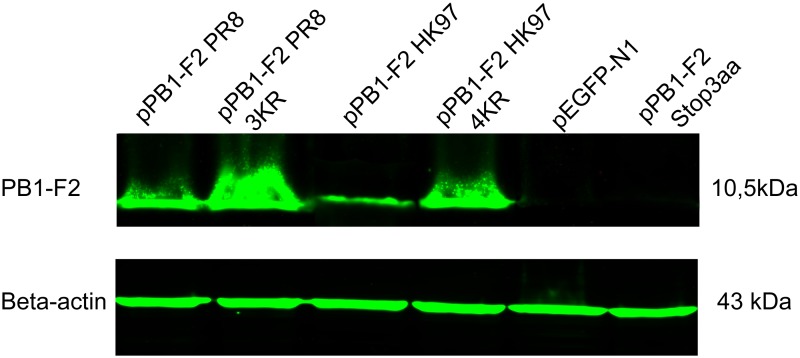
Comparison of the relative expression of the C-terminal cluster K→R mutants of the PR8- and HK97-derived PB1-F2 compared with the wt. 293T cells were co-transfected with pEGFP-N1 (2 ng) and the respective PB1-F2-expressing plasmid (4 μg pPB1-F2 PR8/pPB1-F2 PR8 3KR/pPB1-F2 HK97/ pPB1-F2 HK97 4KR/pPB1-F2 Stop3aa/pEGFP-N1 measured immediately before use). At 24 hr p.t., the transfection efficiency was estimated through epifluorescence. The cells were lysed and the protein concentration was measured. Equal amounts of proteins were loaded and separated through SDS-PAGE. The mAb AG55, specific for the N-terminal portion of PB1-F2, was used for the detection of PB1-F2, and the mAb C15 (Sigma-Aldrich) was used for the detection of Beta-actin. Alexa Fluor 790 Goat anti-mouse IgG (H+L) was used as secondary antibody. The nitrocellulose membrane was scanned using the Odyssey CLx infrared imaging system. Four independent experiments were performed and a representative result is presented.

To determine whether the replacement of the C-terminal K residue cluster of PB1-F2 affects stability and the overall expression of PB1-F2 protein, 293T cells were transfected with equal amounts of plasmid DNA for pPB1-F2 PR8, pPB1-F2 PR8 3KR, pPB1-F2 HK97, pPB1-F2 HK97 4KR, pPB1-F2 Stop3aa, or pEGFP-N1. In addition to different PB1-F2-expressing plasmid, the cells were transfected with 2000-fold less pEGFP-N1. At 24 hr p.t., the transfection efficiency was monitored using fluorescence microscopy. The cells were lysed, and the protein concentration was determined. Equal amounts of protein were subjected to Western blot analysis, in which both beta-actin and PB1-F2 were detected. An N-terminal-specific monoclonal antibody (AG55) was used to avoid any interference in binding to PB1-F2 molecules with of K→R substitutions in the C-terminal K residue cluster. As the binding of AG55 to PB1-F2 HK97 was impaired in immunoblotting analysis, it was not possible to make any comparison between the expression levels of PR8- versus HK97-derived PB1-F2 protein. However, it is possible to compare the relative expression level of wt versus K→R mutants. A significantly higher level of protein expression was detected for the ubiquitination-deficient PB1-F2 PR8 3KR compared with wt PB1-F2 and for HK97-derived PB1-F2 HK97 4KR compared with wt PB1-F2 HK97 (Fig. 5). Importantly the effect of the K→R mutation is not exclusively an artificial phenomenon, as the same effect on protein stability was observed for the p12(I) protein of HTLV-1 [45]. As follows from the immunofluorescence analysis of transfected MDCK cells, the loss of ubiquitination also enhances the expression kinetics of PR8-derived PB1-F2 (S1 Fig.) PB1-F2 is an example of an unstable, short-lived molecule. For PB1-F2 C-terminal K→R mutants, we propose that expression is shifted through the suppression of proteasomal degradation, thereby inducing accumulation. This hypothesis is supported by the earlier detection of PB1-F2 PR8 3KR (S1 Fig.).

### The activity of vRdRp is positively affected by the stabilization of PR8- and HK97-derived PB1-F2

**Fig 6:**
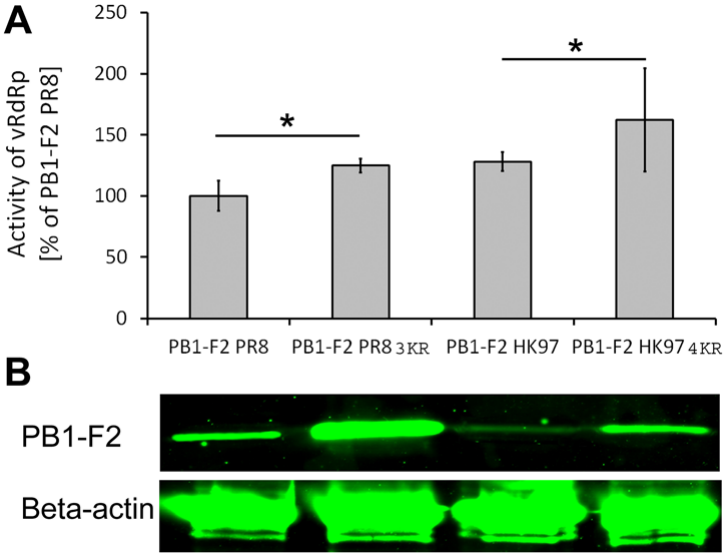
Effect of PB1-F2 stabilization on the activity of vRdRp. (A) A minigenome reporter assay was used for the quantification of the effect of PB1-F2C-terminal K cluster mutation on the activity of vRdRp. The 293T cells were transfected with a set of expression plasmids for the vRdRp subunits pPB1, pPB2, pPA, and pNP, pRLTK (transfection efficiency normalization) and the reporter plasmid pPolPR8MLuc, encoding the firefly luciferase gene under the control of the IAV matrix protein gene segment promoter. Additionally, pPB1-F2 PR8, pPB1-F2 PR8 3KR, pPB1-F2HK97, or pPB1-F2 HK97 4KR plasmids were co-transfected. At 48 hr p.t., the samples were harvested and the activities of luciferases were quantified using a Dual Luciferase Reporter Assay System kit (Promega) on a BIOTEK Synergy HT luminometer. The samples were measured in triplicate and normalized to renilla luciferase activity (transfection efficiency) and firefly luciferase activity (vRdRp activity) in samples co-transfected with pPB1-F2 PR8. The data represent 3 independent experiments. The bars indicate the average value of the independent experiments, and the error bars indicate the intra-experiment SD values. The asterisk indicates a significant difference between the groups compared (*P<0*.*05*). (B) PB1-F2 protein and Beta-actin were detected using western blotting to compare the relative activity of vRdRp with the relative amount of the PB1-F2 in the same samples.

The effect of PB1-F2 on the activity of vRdRp is complex, depending on the presence of specific amino acids, the virus strain and the host cell type infected. Nevertheless, studies have consistently shown that PB1-F2 increases vRdRp activity [6–8, 46]. The effect of PB1-F2 on vRdRp is not surprising, as the interaction between the vRdRp catalytic subunit PB1 and PB1-F2 has been widely accepted [4, 5]. The subcellular localization of PB1-F2 influences the activity of vRdRp. The localization of avian PB1-F2 to the nucleus has been associated with enhanced RdRp activity, while mitochondria-targeted PB1-F2 variants affect polymerase activity to a lower extent [6, 8, 47]. A recent report suggested that the presence of PB1-F2 ORF is a minimal, but not satisfactory prerequisite for PB1-F2 expression. Internal mRNA regulatory elements could affect the PB1-F2 expression rate [27]. Based on these results, we determined whether the increased stability of PB1-F2 correlates with the enhanced activity of vRdRp. We constructed a reporter plasmid encoding firefly luciferase under the control of the IAV promoter sequence of the matrix protein gene segment. Human embryonic kidney cells (293T) were transfected with the expression plasmids pPB1, pPB2, pPA, or pNP (encoding vRdRp) along with the transfection efficiency normalization plasmid pRLTK, a vRdRp activity reporter plasmid, and different PB1-F2-expressing plasmids (pPB1-F2 PR8/pPB1-F2 PR8 3KR/pPB1-F2 HK97/ pPB1-F2 HK97 4KR/78/85R). At 48 hr p. t., the activity of vRdRp was measured and normalized to renilla luciferase activity. Three independent experiments were performed in triplicate for each sample. The results were further normalized to the activity of vRdRp in the presence of PR8-derived PB1-F2 (Fig. 6). As expected, the activity of vRdRp in the presence of avian PB1-F2 HK97 was higher than in the presence of PB1-F2 PR8. The activity of vRdRp in the presence of stabilized PB1-F2PR8 3KR was nearly equal to that of PB1-F2 HK97 and increased 25% compared with PB1-F2 (Fig. 6A). Similarly, the stabilization of HK97-derived PB1-F2 after K73/78/81/85R mutation enhanced vRdRp activity 34, 5% compared with PB1-F2 HK97, and this effect is approximately > 62% higher than vRdRp activity in presence of PB1-F2 PR8. To exclude the possibility that K→R mutations in the PR8- or HK97-derived PB1-F2 ORF are directly responsible for vRdRp activity, the same samples in which vRdRp activity was measured were subjected to immunoblot detection to determine PB1-F2 expression levels. Beta-actin was co-stained for normalization. Despite impairment in AG55 binding to PB1-F2 HK97 and the fact that it is not possible to associate the expression levels of PR8- versus HK97-derived PB1-F2 proteins, (PR8/HK97) K→R mutations in the C terminal K residue cluster enhanced PB1-F2 expression (Fig. 6B), suggesting that the stabilization of the PB1-F2 affects vRdRp activity, regardless of PB1-F2 virus origin.

### PB1-F2 PR8 3KR exhibits an enhanced antibody response, while wt PB1-F2 is a weak antibody inducer

**Fig 7:**
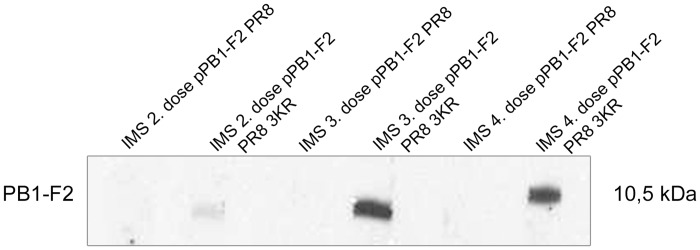
Antibody responses to pPB1-F2 PR8 vs. pPB1-F2 PR8 3KR after DNA immunization. The mice (n═ 5) were immunized four times at two-week intervals with 50 μg of pPB1-F2 PR8 or pPB1-F2 PR8 3KR DNA. MDCK cells were transfected with pPB1-F2 PR8 and lysed directly in the wells at 24 hr p. t. using SDS sample buffer. The proteins were separated through SDS-PAGE in a single well and transferred to nitrocellulose membrane. The sera were pooled after each dose and assayed for the presence of anti-PB1-F2 antibodies using immunoblotting on a sliced nitrocellulose membrane.

PB1-F2-specific antibodies are induced through different IAV strains in mice and humans, but multiple infections with heterologous viruses are needed to reach a detectable antibody response [24, 25]. The immunogenicity of any antigen is a complex function affected by several factors, including molecular size, chemical composition, heterogeneity, susceptibility to processing and presentation, glycosylation and dosage. PB1-F2 is weakly immunogenic; however, we recently published results indicating the protection potential of anti-PB1-F2 antibodies [26]. As the effect of K73, 78, and 85R substitutions are coupled with intracellular UPS, DNA vaccination represents a reasonable approach for studying potentially altered immunogenicity. The mice (n═5) were immunized four times i.m. with pPB1-F2 PR8 or pPB1-F2 PR8 3KR at two-week intervals. The samples were obtained after combining the serum samples collected at particular intervals of immunization. Because ELISA using the lysate of cells transiently transfected with pPB1-F2 PR8 did not provide satisfactory results, the antibodies were used for detected through Western blotting. MDCK cells were transiently transfected with pPB1-F2 PR8, and the cell lysates were separated through SDS-PAGE, followed by blotting onto nitrocellulose membranes. The membrane was cut and the pooled immune mouse sera (100-fold diluted) were incubated with the membrane slices to detect anti-PB1-F2 antibodies (Fig. 7). In pPB1-F2 PR8 DNA immunized mice, no detectable anti-PB1-F2 response was observed, even after a fourth dose of DNAv. In contrast, the mice receiving pPB1-F2 PR8 DNA immunization showed detectable levels of anti-PB1-F2 antibodies as early as 14 days after the second DNAv dose of pPB1-F2 PR8 3KR. Notably, a third dose led to a significant increase in the antibody levels, while further immunization with pPB1-F2 PR8 3KR did not significantly alter the levels of anti-PB1-F2 antibodies. We demonstrated that the inhibition of ubiquitination increases PB1-F2 accumulation. Genetic fusion of the tuberculosis protein MTP64 with UBQ resulted in enhanced degradation *in vitro*. DNA immunization with the fusion constructs lead to a strong TH1 response, but no detectable humoral response [48]. Similar observations were also published for the LCMV NP antigen. The co-translational addition of UBQ abrogated the detectable antibody response, while the CTL response was significantly improved [49]. These authors suggest that rapid degradation interferes with BCR recognition. However, it is not surprising that the genetic attachment of UBQ does not typically interfere with the humoral response [50, 51] because balanced degradation is needed to properly induce humoral and cellular immune responses to particular proteins. In contrast with the genetic fusion of UBQ to enhance antigen processing, we employed a different approach to the genetic blocking of ubiquitination. The alternation of the UBQ acceptor sites on PB1-F2 facilitated the characterization of the relationship between ubiquitination and the stability and induction of the humoral response to PB1-F2. Ubiquitination is directly involved in proteasome-mediated protein degradation and MHC class I peptide presentation and virus recognition. Several integral IAV proteins, including NP, M1, M2, PB1, and NS1 induce an effective antiviral CTL response (extensively reviewed) [52]. A recent study on MHC I haplotype D^b^ mice suggested that the reduced cytotoxicity and accelerated decay of the TCR-MHCI peptide complex occurs in PB1-F2-specific CTLs compared with NP- and PA-specific CTLs. However, this subject was beyond the scope of the present study. The modulation of the PB1-F2 CTL response through blocking ubiquitination should not be excluded.

### IFN-β antagonism is enhanced by stabilization of PR8 as well as HK97 derived PB1-F2

**Fig 8:**
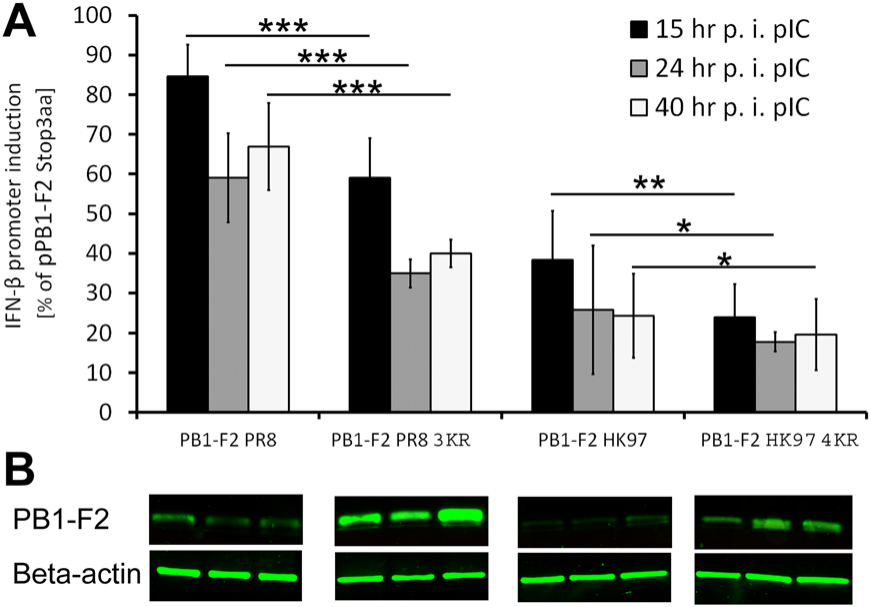
Effect of PB1-F2 stabilization on the induction of the human IFN-β promoter. (A) A 293T cells were co-transfected with the human IFN-β promoter reporter plasmid pGLhIFNBpr, pRLTK (transfection efficiency normalization) and the respective PB1-F2 expressing plasmids (pPB1-F2 PR8/pPB1-F2 PR8 3KR/pPB1-F2 HK97/pPB1-F2 HK97 4KR/pPB1-F2 Stop3aa). At 24 hr p.t., the IFN-β promoter was further induced through the addition of pIC. At 15, 24 and 40 hr p.t., the supernatant was removed, and the cells were gently washed, directly lysed using passive lysis buffer (Promega) and the luciferase activity was quantified using the Dual Luciferase Reporter Assay System kit (Promega) on a BIOTEK Synergy HT luminometer. The samples were measured in triplicate and normalized to renilla luciferase activity (transfection efficiency) and firefly luciferase activity (human IFN-β promoter induction) determined for pPB1-F2 Stop3aa co-transfected samples. The data represent 3 independent experiments. The bars indicate the average value of the independent experiments, and the error bars indicate the intra-experiment SD values. The asterisk indicates a significant difference between the groups compared (* *P<0*.*05*; ** *P<0*.*01*; *** *P<0*.*001*). (B) PB1-F2 protein and Beta-actin was detected through western blotting to compare the induction of IFN-β promoter with the relative amount of the PB1-F2 in the same samples.

Immediately after IAV invades the host organism, the cells establish a robust antiviral state mediated through IFN-α/β, which collectively labels the IFN I response. After PAMPs (ssRNA, dsRNA) are recognized by PRRs (TLR, RIG-I), IFN-β is massively produced [53, 54]. The IFN-α and IFN-β genes are directly induced in response to viral infection [55]. While IFN-β is expressed by most cell types in response to viral infection or other stimuli, plasmacytoid dendritic cells are likely the predominant cells expressing IFN-α [56]. Because IAV initially infects respiratory epithelial cells, macrophages and dendritic cells, we focused on IFN-β expression in response to PB1-F2. PB1-F2 acts antagonistically to the IFN response, most likely through interactions with the mitochondrial antiviral protein MAVS [13, 17, 18]. In contrast, the PB1-F2-mediated enhancement of IFN-β expression in the presence of other IAV proteins has also been reported [57], suggesting that the observed effect is not solely attributed to PB1-F2. The IAV IFN antagonist NS1 protein acts through different mechanisms compared with PB1-F2. Additionally, the PB2 subunit of vRdRp localizes to mitochondria and interacts with the MAVS protein to inhibit IFN-β [58]. To assess the connection between the expression levels of PB1-F2 and the induction of the IFN-β promoter, we performed luciferase reporter assays (Fig. 8). The 293T cells were co-transfected with the human IFN-β promoter firefly luciferase reporter, a plasmid constitutively expressing renilla luciferase and pPB1-F2 PR8/pPB1-F2 PR8 3KR/pPB1-F2 HK97/pPB1-F2 HK97 4KR plasmids. At 24 hr p.t., the IFN-β pIC inducer was added to the cells, and IFN-β promoter induction was measured at 15, 24, and 40 hr p.i. (Fig. 8A). The results were normalized to renilla luciferase activity for transfection efficiency and cytotoxicity normalization. Further normalization was applied to IFN-β promoter induction in cells co-transfected with pPB1-F2 Stop3aa. The samples were simultaneously tested via immunoblotting to detect the relative amount of PB1-F2 and Beta-actin protein. Although AG55 binding to PB1-F2 HK97 is impaired and it is not possible to relate the expression levels of PR8- versus HK97-derived PB1-F2 proteins, immunoblotting confirmed the increased expression of stabilized PR8- and HK97-derived PB1-F2 variants (Fig. 8B). In both cases, the stabilization of PB1-F2 by the K→R mutation in the C-terminal K residue cluster enhanced IFN-β promoter inhibition. A comparison of the samples transfected with pPB1-F2 HK97 to pPB1-F2 HK97 4KR revealed only a slight enhancement of inhibition (15%-15, 8%-24, 4,7%-40 hr p.i. pIC) due to PB1-F2 stabilization. At forty hours post-transfection pIC, there was no significant difference between these two groups, suggesting the early inhibition of IFN-β promoter, consistent with previous studies [13]. Stronger inhibition of IFN-β promoter was observed between pPB1-F2 PR8- and pPB1-F2 PR8 3KR-transfected samples (25, 6%-15, 25%-24, 26%-40 hr p.i. pIC). However, PB1-F2 HK97 was a much more potent antagonist of IFN-β compared with PB1-F2 PR8 protein (Fig. 8A). Although it has previously been shown that HK97-derived PB1-F2 inhibits the early IFN response [13], it has ben suggested that the IFN antagonism of PB1-F2 (PR8/HK97) is mediated via mitochondrial membrane protein MAVS. Notably, it has also been shown that PR8-derived PB1-F2 colocalizes with MAVS in mitochondria [17]. Because the colocalization of PB1-F2 HK97 and the stabilized K→R variant was not observed (Fig. 4), it is surprising that HK97-derived PB1-F2 inhibition of the IFN-β promoter is stronger than PR8-derived PB1-F2 inhibition. This observation suggests another MAVS-independent mechanism for HK97-derived PB1-F2 IFN antagonism. However, a recent study showed that for avian H5N1- and H7N9-, but not for mammalian H1N1 (PR8), virus-derived PB1-F2 IFN-β inhibition is mediated via the regulation of NF-kB signaling [59].

## Conclusion

Insufficient knowledge is available on the role of the posttranslational modification of PB1-F2 in relation to the biological functions of this protein. Herein, we provided direct evidence for the posttranslational modification of PB1-F2 through ubiquitination. We identified a cluster of lysine residues for ubiquitin binding. The mutant variant PB1-F2 with C-terminal K residue cluster replaced by R facilitated the characterization of a crucial role for PB1-F2. Positively affected PB1-F2 stability enhances vRdRp activity and PB1-F2-mediated IFN antagonism. Similar effects of changes in the C-terminal K residue cluster was observed for evolutionary distinct avian A/Honk Kong/156/1997 (H5N1)- and mammalian A/Puerto Rico/8/1934 (H1N1)-derived PB1-F2, suggesting general role for the C-terminal K residue cluster in PB1-F2 stability. As the lysine cluster on the C-terminal portion of PB1-F2 is highly conserved, it will be critical to characterize the biological properties of the PB1-F2 K→R-expressing influenza A virus.

## Supporting Information

S1 Fig Effect of K73/78/85R mutation of PB1-F2 on expression kinetics. MDCK cells were transfected with 1 μg of pPB1-F2 PR8 DNA (upper rows) or pPB1-F2 PR8 3KR DNA (lower rows). At the indicated post-transfection times, the samples were fixed, permeabilized and the relative levels of PB1-F2 expression (green) were detected using the PB1-F2 N-terminal specific mAb AG55 and a secondary FITC conjugated antibody. The samples were analyzed using a Zeiss LSM 510 Meta confocal microscope. All images were acquired under the same conditions.
(TIF)
