# Supplementary figures and images for: The Ubiquitination of the Influenza A Virus PB1-F2 Protein Is Crucial for Its Biological Function
Source: PLoS One. 2015 Apr 13;10(4):e0118477. doi: 10.1371/journal.pone.0118477 (PMC4395099; doi:10.1371/journal.pone.0118477)

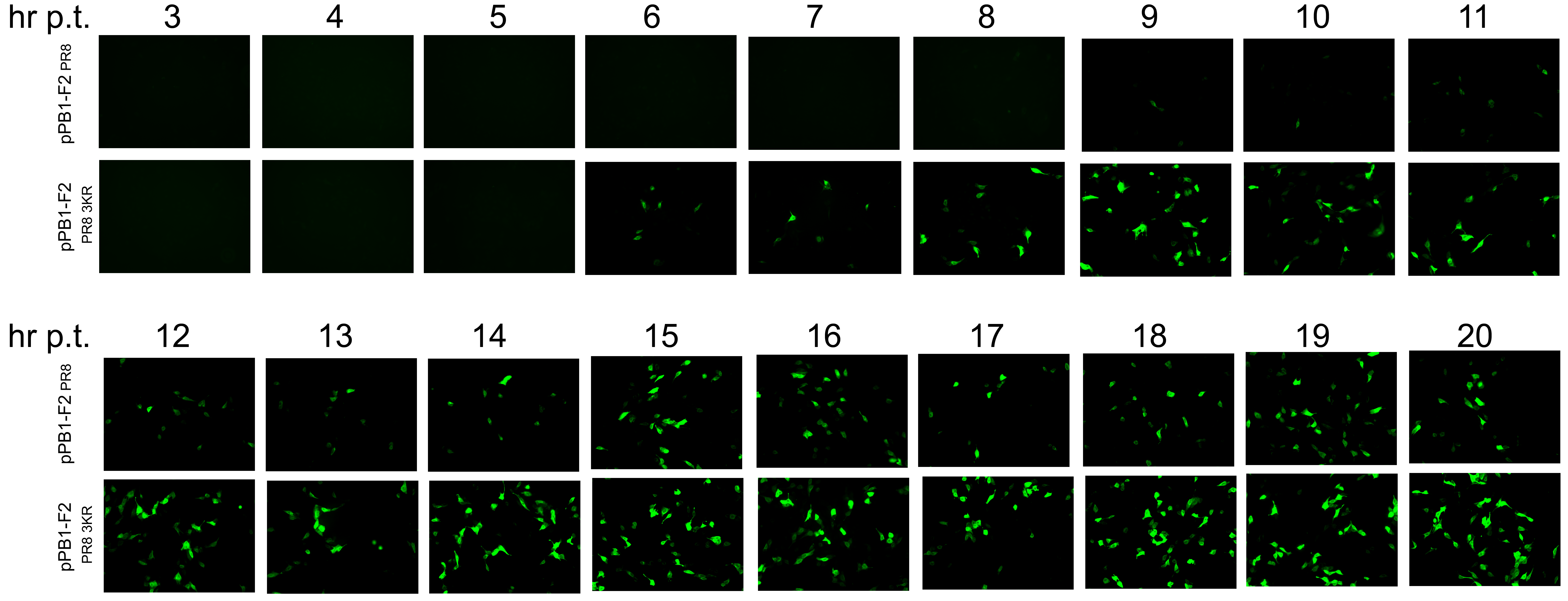

Supplement: S1 Fig — MDCK cells were transfected with 1 μg of pPB1-F2 PR8 DNA (upper rows) or pPB1-F2 PR8 3KR DNA (lower rows). At the indicated post-transfection times, the samples were fixed, permeabilized and the relative levels of PB1-F2 expression (green) were detected using the PB1-F2 N-terminal specific mAb AG55 and a secondary FITC conjugated antibody. The samples were analyzed using a Zeiss LSM 510 Meta confocal microscope. All images were acquired under the same conditions. (TIF) [file pone.0118477.s001.tif]
